# Classification of Osteophytes Occurring in the Lumbar Intervertebral Foramen

**DOI:** 10.3390/tomography10040047

**Published:** 2024-04-19

**Authors:** Abdullah Emre Taçyıldız, Feyza İnceoğlu

**Affiliations:** 1 Department of Neurosurgery, Faculty of Medicine, Turgut Özal University, Malatya 44090, Turkey; 2 Department of Neurosurgery, Faculty of Medicine, Karabuk University, Karabuk 78200, Turkey; 3 Department of Biostatistics, Faculty of Medicine, Turgut Özal University, Malatya 44090, Turkey; feyza.inceoglu@ozal.edu.tr

**Keywords:** foraminal stenosis, lumbar intervertebral foramen, osteophyte

## Abstract

Background: Surgeons have limited knowledge of the lumbar intervertebral foramina. This study aimed to classify osteophytes in the lumbar intervertebral foramen and to determine their pathoanatomical characteristics, discuss their potential biomechanical effects, and contribute to developing surgical methods. Methods: We conducted a retrospective, non-randomized, single-center study involving 1224 patients. The gender, age, and anatomical location of the osteophytes in the lumbar intervertebral foramina of the patients were recorded. Results: Two hundred and forty-nine (20.34%) patients had one or more osteophytes in their lumbar 4 and 5 foramina. Of the 4896 foramina, 337 (6.88%) contained different types of osteophytes. Moreover, four anatomical types of osteophytes were found: mixed osteophytes in 181 (3.69%) foramina, osteophytes from the lower endplate of the superior vertebrae in 91 (1.85%) foramina, osteophytes from the junction of the pedicle and lamina of the upper vertebrae in 39 foramina (0.79%), and osteophytes from the upper endplate of the lower vertebrae in 26 (0.53%) foramina. The L4 foramen contained a significantly higher number of osteophytes than the L5 foramen. Osteophyte development increased significantly with age, with no difference between males and females. Conclusions: The findings show that osteophytic extrusions, which alter the natural anatomical structure of the lumbar intervertebral foramina, are common and can narrow the foramen.

## 1. Introduction

The lumbar intervertebral foramen (IVF) comprises two movable joints and a complex three-dimensional (volumetric) structure that few surgeons understand [1,2]. The lumbar IVF contributes to the development of radiculopathy and its surgical treatment [2].

In general, osteophytes are a feature of osteoarthritis and are classified as extraspinal or vertebral [3]. The pathophysiology of osteoarthritis involves the proliferation of periosteal cells at the bone–cartilage border. Mechanical stimuli are the most likely cause of this proliferation [3]. TGF-β and morphogenetic protein 2 play critical roles in osteophyte formation [3].

Back pain is a personal and societal burden that significantly reduces the quality of life worldwide; it causes severe disability and requires substantial healthcare resources [4,5]. Significant evidence suggests a relationship between the severity of low back pain and the presence of spinal osteoarthritis and disk space narrowing [6]. Osteoarthritis in the lumbar spine causes low back pain and lowers the quality of life [7]. Low back pain is not observed in people without degenerative osteoarthritis, as confirmed through lumbar magnetic resonance images [7]. Obesity, lumbar disk degeneration, and spinal osteoarthritis contribute to spinal degeneration [8].

Foraminal stenosis is one of the most common findings (25–29%), and it is used to diagnose failed back surgery syndrome [9]. Previous studies have focused on the intervertebral foramen—a critical area for surgeons—and its morphology and anatomy [1,2]. However, a literature review revealed a lack of research on foraminal osteophytic pathologies in the sagittal plane [10]. The present study aims to visualize the location of osteophytes in the intervertebral foramen. The study also identifies the frequency, anatomical regions, and distribution of the osteophytes involved in lumbar IVF. Another aim is to statistically present the presence of osteophytes based on age and gender. This study aims to detect pathologies in this region, provide a clinical guideline, and contribute to biomechanics research. Foraminal space narrowing, osteoarthritic processes, and the distribution of osteophytes in the intervertebral foramina are important factors to consider when performing surgery and conducting biomechanical studies. Understanding lumbar IVF osteophytes could help us better understand one of the causes of low back pain.

## 2. Materials and Methods

### 2.1. Patient Population

Our study is a retrospective, non-randomized, single-center study involving 1224 patients who presented to the Faculty of Medicine, Malatya Turgut Özal University, between 1 January and 31 December 2021. The data from patients who were asked to undergo computed tomography (CT) of the lower abdomen after visiting the emergency department, urology clinic, or general surgery clinic were reviewed retrospectively. CT scans of the lower abdomen were reconstructed in the bone window using the PACS v4.1.2.40 software from our hospital’s electronic database. Moreover, the L4 (L4–L5) and L5 (L5–S1) intervertebral foramina were evaluated bilaterally. The gender, age, and anatomical location of the osteophytes in the lumbar intervertebral foramina of the patients were recorded. This retrospective study was approved by the Ethics Committee of Clinical Research of the Faculty of Medicine, Malatya Turgut Özal University, with decision no. 2021/111, dated 16 December 2021.

### 2.2. Inclusion and Exclusion Criteria

Patients who had undergone a neurosurgical procedure and those for whom neurosurgeons requested lumbar CT scans were excluded from this study. Moreover, patients whose post-reconstruction images had visibility issues that interfered with interpretation were excluded from the study. Patients under 18 years old were not allowed to participate.

### 2.3. Image Analysis and Research Methods

The sagittal plane was used to examine the reconstructed lumbar vertebrae. Foramina L4 and L5 were evaluated bilaterally by a single observer. The sagittal section was evaluated until both foramina exited, allowing visualization of the right and left sides (until the sagittal sections were completed). The natural anatomical boundaries of the evaluated lumbar vertebrae were determined using standard atlases of human anatomy [11]. The osteophytes were defined as extrusions from the natural anatomical boundaries of the lumbar vertebrae and classified based on the anatomical region in which they originated from the lumbar vertebrae (i.e., the junctions of the pedicles and laminae, the upper endplate of the lower vertebrae, and the lower endplate of the upper vertebrae). If they extruded from multiple regions, they were classified as a mixed type.

The imaging equipment included Philips Ingenuity CT, 2014, 128 slices, serial no. 600021 (Philips Healthcare, Eindhoven, The Netherlands) and Philips MX, 2014, 16 slices, serial no. EP16E140004 (Philips Healthcare, Eindhoven, The Netherlands). The slice thickness (for both CT machines) was 2.5 mm. First, axial sections were created. The image was then reconstructed into sagittal sections using the PACS v4.1.2.40 software.

### 2.4. Statistical Analysis

The data from this study were analyzed using the statistical program in the Social Sciences 25 software. The Shapiro–Wilk test was used to determine whether the data from this study followed a normal distribution. For comparison tests, a significance level (*p*) of 0.05 was used. Because the variables did not follow the normal distribution (*p* > 0.05), the analysis was continued using nonparametric tests. The Mann–Whitney U test was used for independent paired group comparisons because the normality assumptions were not met. The Kruskal–Wallis test was used to compare multiple independent groups. As the number of comparisons between the variables showing differences increased, the Bonferroni corrected *p*-value was used and calculated as 0.05 (binary comparison). This study had four groups and two comparisons, resulting in the following calculation: = 6, *α*_BD_ = 0.05/6 = 0.008. After the Kruskal–Wallis test was conducted, the *p*-values obtained from the Mann–Whitney U test were compared with the value of 0.008, and the results were determined. To analyze the categorical data, we created cross tables and used chi-square (*χ*^2^) analysis.

## 3. Results

In this study, 1224 cases and 4896 lumbar intervertebral foramina were examined. Of the cases, 527 (43.1%) were females and 697 (56.9%) were males. The mean age of the participants was 47.75 ± 19.03 years. Among the study participants, the highest age was 109 years, and the lowest age was 18 years (Table 1). The lowest ages at which osteophytes were discovered were 19 and 20 in 1 and 3 patients, respectively. One or more osteophytes were found in the lumbar 4 and 5 foramina in 249 (20.34%) patients.

**Figure 1:**
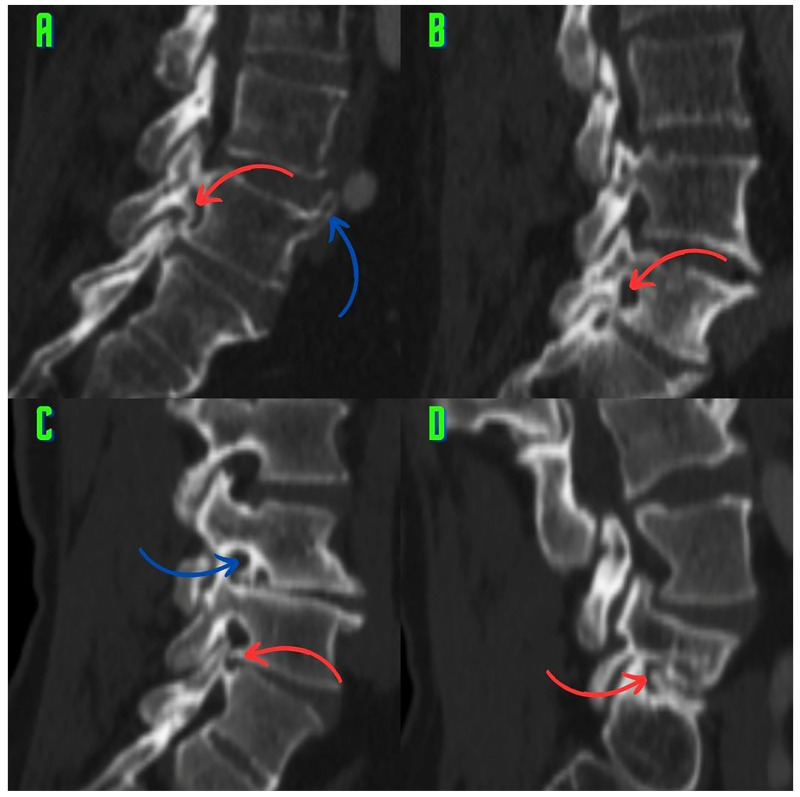
Mixed-type osteophytosis is observed. (**A**) An osteophyte (red arrow) arising from the pedicle–lamina junction and the inferior endplate of the superior vertebrae is observed in the L5–S1 foramen. Vertebral osteophytes (blue arrow) are also observed at a position anterior to the vertebrae. (**B**) Osteophytes (red arrow) arising from three different points united in the foramen. A mixed-type osteophyte arising from the pedicle–lamina junction, lower endplate of the superior vertebrae, and upper endplate of the inferior vertebrae formed an interesting pathoanatomical structure in the foramen. (**C**) A mixed-type osteophyte (blue arrow) arising from the pedicle–lamina junction in the L4–5 foramen and an osteophyte (red arrow) arising from the lower endplate of the superior vertebrae in the L5–S1 foramen are observed. (**D**) The L5–S1 disk space appears to have collapsed. A mixed-type osteophyte (red arrow) is seen to be arising from both the lower endplate of the superior vertebrae and the upper endplate of the inferior vertebrae. The fact that the spines are not in the same plane in sagittal images gives the impression of degenerative scoliosis.

**Figure 2:**
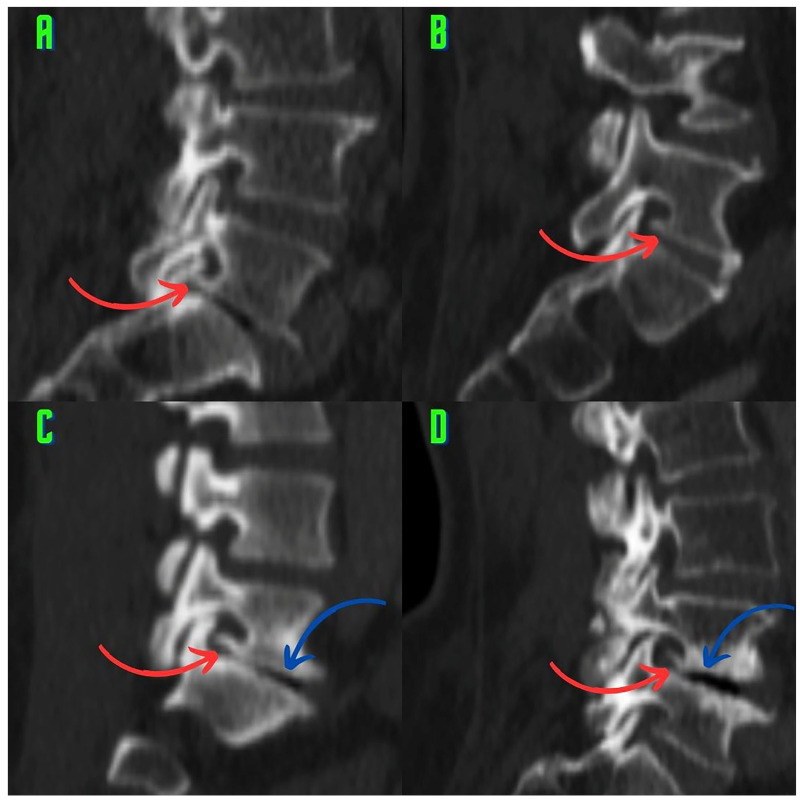
Osteophytes arising from the lower endplate of the superior vertebrae are observed. (**A**) An osteophyte arising from the lower endplate of the superior vertebrae (red arrow) in the L5–S1 foramen. It is observed that the intervertebral foramen is narrowed. (**B**) An osteophyte arising from the lower endplate of the superior vertebrae (red arrow) in the L5–S1 foramen. (**C**) In the L5–S1 foramen, the osteophyte arising from the inferior endplate of the superior vertebrae (red arrow) almost merges with the superior articular facet. The disk space is narrowed, and the vacuum phenomenon (blue arrow) is observed. (**D**) An osteophyte (red arrow) extending cranially from the inferior endplate of the superior vertebrae potentially narrows the foraminal space. Vacuum phenomenon (blue arrow) is observed.

**Figure 3:**
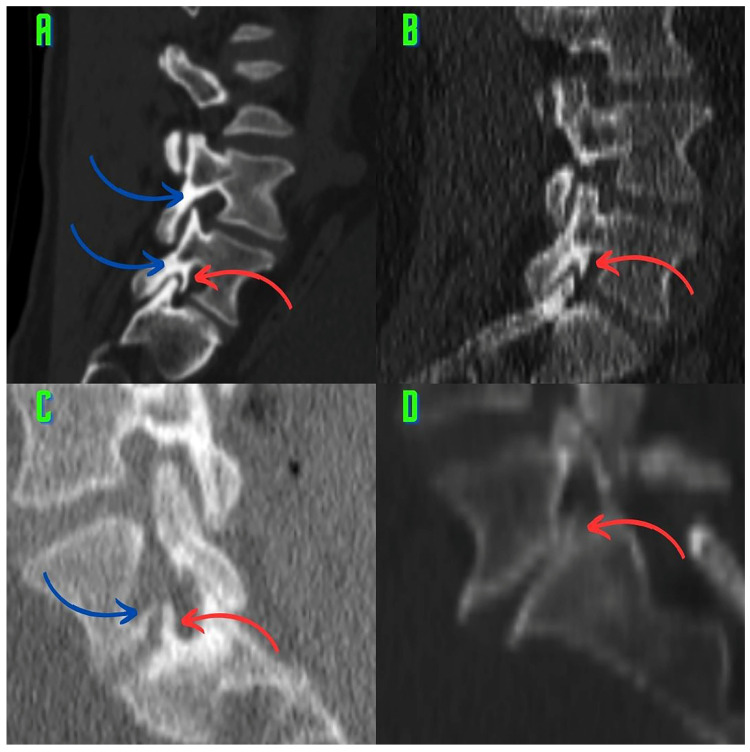
In the L5 (L5–S1) foramina, osteophytes arising from the pedicle–lamina junction and osteophytes arising from the superior endplate of the lower vertebrae are observed. (**A**) An osteophyte (red arrow) is observed in the L5–S1 foramen originating from the pedicle–lamina junction and extending to the anterior of the superior articular process. Hyperostosis (blue arrow) is observed in the pars region. (**B**) An osteophyte (red arrow) arising from the pedicle–lamina junction is observed in the L5–S1 foramen. (**C**) An osteophyte (red arrow) arising from the lower vertebral endplate of the intervertebral foramen is observed. Moreover, an osteophyte (blue arrow) arising from the lower endplate of the superior vertebrae is observed. (**D**) An osteophyte (red arrow) arising from the superior endplate of the lower vertebrae in the L5–S1 foramen exhibits oblique extension and occupies the foramen.

**Figure 4:**
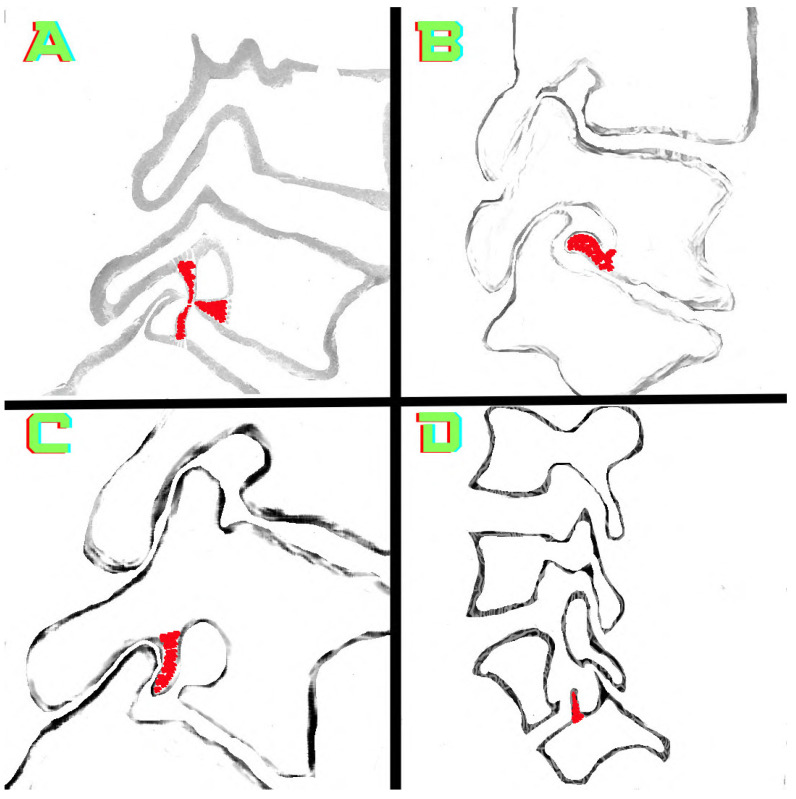
Intervertebral foramina and osteophytes are illustrated. (**A**) Osteophytes (red area) arising from three different points. (**B**) An osteophyte (red area) arising from the inferior endplate of the superior vertebrae. (**C**) An osteophyte (red area) arising from the pedicle–lamina junction. (**D**) A big osteophyte (red area) arising from the lower vertebral endplate of the intervertebral foramen is observed. Simultaneously, osteophytization is observed at two distinct points.

Various types of osteophytes were found in 337 (6.88%) of the 4896 foramina. Moreover, mixed-type osteophytes were found in 181 foramina (3.69%, Figure 1), and osteophytes extruded from the lower endplate of the superior vertebrae in 91 foramina (1.85%, Figure 2), from the junction of the pedicle and lamina of the upper vertebrae in 39 foramina (0.79%, Figure 3A,B), and from the upper endplate of the lower vertebrae in 26 (0.53%) foramina (Figure 3C,D). Overall, 248 and 89 osteophytes were found in the L5 and L4 intervertebral foramina, respectively. Four different types of osteophytes were identified (Figure 4).

There was a significant difference between the measurements of the L5 and L4 foramina in the participants. The L5 foramen had a higher incidence of osteophytes than the L4 foramen (*p* < 0.05, Table 2). Among the participants included in the study, there was no statistically significant difference between the males and females in terms of the measurements of the right L5–S1, the left L5–S1, both the right and left L5–S1, the right L4–L5, the left L4–L5, and both the right and left L4–L5 (*p* > 0.05, Table 3). The osteophyte formations in the foramen increased significantly with age (*p* < 0.05, Table 4).

## 4. Discussion

This study presented descriptive statistics and visual evidence for the morphology and distribution of lumbar foraminal osteophytes. The study also allowed a discussion of various points of view on the potential effects of lumbar foraminal osteophytes. To the best of our knowledge, there are no similar studies in the literature, which made making comparisons challenging at some points.

This study suggests that classifying the different types of osteophytes can play a critical role in the planning of surgical interventions. Treatment approaches that are tailored to the locations of the osteophytes can significantly improve patients’ recovery processes. This information enables surgeons to select more accurate intervention methods and to reduce the risk of potential complications. Accurately identifying osteophytes (e.g., the lower endplates of the upper vertebrae) can improve the effectiveness of targeted surgical interventions and result in significant pain relief for the patient (Figure 3C,D and Figure 4D). Our findings provide visual evidence of foraminal stenosis (Figure 1, Figure 2, Figure 3 and Figure 4). Additionally, this classification system can be used as an effective teaching tool in spinal surgery training programs (Figure 1, Figure 2, Figure 3 and Figure 4). Young surgeons can use this information to better understand the anatomical variations of osteophytes and their potential clinical implications. This classification provides a foundation for a more in-depth study of osteophytes and their clinical outcomes. Future research can contribute to the development of customized approaches in spinal surgery by evaluating the impact of this classification on surgical outcomes in greater detail.

The study results provide a detailed map of the foraminal osteophytes. Four osteophytes were identified based on their anatomical locations in the L4–L5 and L5–S1 foramina (Figure 1, Figure 2, Figure 3 and Figure 4). Lumbar spinal osteophytes are more prevalent in the L5 (L5–S1 level) foramen (Table 2). The presence of these osteophytes does not vary by gender (Table 3), and age plays an important role in osteophyte formation (Table 4). This finding is consistent with those reported in the relevant literature. Previous research has found that osteophytes increase with age in other anatomical regions of the body (such as the knees) [3].

### 4.1. Osteophytes and Facet Osteoarthritis

Facet osteoarthritis is recognized as a cause of severe low back pain, affecting the economy through lost labor and, more importantly, health. Some studies have found that the facet joint and osteoarthritis can be the source of pain in these patients [12,13]. Facet osteoarthritis is a pathological condition characterized by degenerative and proliferative processes, such as subarticular bony erosions, joint space narrowing, articular process hypertrophy, osteophytosis, and an imbalance between destruction and repair [14]. Previous research has shown that facet osteoarthritis is more common at the L4–L5 level [15]. Our study revealed that osteophyte rates were higher in the L5 foramen (i.e., at the L5–S1 level; *p* < 0.05, Table 2). This finding is also consistent with the findings in the existing literature. Although osteophytes and osteoarthritis are correlated, they are different concepts [3]. Therefore, the presence of more osteophytes in the L5 (L5–S1 level) foramen than in the L4 (L4–L5 level) foramen can be explained by the increased interaction of weight-bearing and other forces in the spine from top to bottom [3,16]. This could be because osteophytes form in response to biomechanical stimuli, as reported in the literature [3].

An imbalance in load distribution is considered to be the primary cause of facet osteoarthritis [17]. The three joints in the motion segment are functionally related [10]. Biomechanical studies have shown that the lumbar disk and two facet joints work together to carry loads [18,19]. Moreover, facet osteoarthritis has a close pathological relationship with lumbar disk degeneration [14,15]. Autopsy studies revealed that facet joint degeneration is always associated with disk degeneration. The autopsy study of Vernon-Roberts and Pirie found that disk degeneration was almost always associated with osteophyte formation in the vertebral margins [20]. As a result, it is widely known that the three segments of motion (the lumbar disk and two facet joints) interact and can degenerate together [10,14,15,18,19]. In our study, disk degeneration accompanied foraminal osteophytes in many cases (Figure 1B–D, Figure 2A,C,D and Figure 3D). Notably, the visual findings of our study support and contribute to the existing literature. Moreover, vertebral osteophytes (Figure 1B and Figure 2A,C,D) are another indicator of disk degeneration and commonly coexist with foraminal osteophytes [3]. At the foraminal region, the parts of the triple motion system are most closely related [1]. Therefore, osteophytes, for which visual evidence was provided in the present study, are candidates for strong radiographic markers of facet osteoarthritis because they represent a pathoanatomical structure that may affect the musculoskeletal system.

Facet osteoarthritis and motion segment failure are thought to contribute to degenerative spondylolisthesis and scoliosis [21]. The visual evidence of the present study supports the literature on the subject. In this study, we presented foraminal osteophytes with sagittal sections, and some cases had concomitant degenerative scoliosis (Figure 1D). According to previous studies, sagittal sections can reveal deformity in degenerative scoliosis [22].

Atul Goel linked the development of facet joint osteophytes to degeneration and instability in this region [23,24]. The visual findings of our study, particularly the signs of degeneration associated with osteophytes (decreased disk height, vertebral osteophytes, and the vacuum phenomenon), are consistent with those of previous studies (Figure 1A,C and Figure 2A,C,D) [23]. The periosteal reaction is thought to be responsible for osteophyte-induced degeneration [24]. It is also noted that these degenerative osteophytes that form around the facet joint narrow the intervertebral neural foramen [24]. Our findings are consistent with those of Atul Goel, who found visual evidence that osteophytes cause foraminal stenosis (Figure 1, Figure 2, Figure 3 and Figure 4) [24].

### 4.2. Potential Effect of Osteophytes on Biomechanics

The findings of the present study may have significant biomechanical and kinematic implications. Kozanek et al. [25] found that in asymptomatic participants, the movement of the facet joints during the flexion–extension movement of the L4–L5 segment was less than that of the upper lumbar segments. In general, they found that the flexion and extension movements were more limited at the lower lumbar levels, whereas the torsion and lateral bending movements were more limited at the upper lumbar levels [25]. These movements involve both the lower lumbar spine, which is coronal and horizontal, and the upper lumbar spine, which is sagittal and vertical [25]. The orientation of the facets directs and limits the movements of the spine. Thus, the orientation aims to reduce mechanical forces while protecting the annulus fibrosus cells from overstretching [16]. Kozanek et al. [25] found that the lower lumbar facet joints limit flexion and extension movements. Moreover, Wilke [26] and Nachemson et al. [27] found that intradiscal pressure is higher in the flexion positions of the spine (i.e., the flexion position while sitting, standing, and lifting weights in the standing position) than in other positions. Mechanical stress is thought to be the first event that causes the formation of osteophytes [3]. We believe that mixed-type osteophytes (Figure 1) and extrusions that develop into osteophytes behind the superior facet at the pedicle–lamina junction (Figure 1 and Figure 3A,B) are modular (regionally independent) responses that aim to further restrict (even immobilize) lumbar flexion and extension movements. Atul Goel [28] suggests that the formation of osteophytes around the facet joint may provide protection by reducing instability. Our modular response view is consistent with Atul Goel’s thoughts. There are three explanations for this viewpoint. First, the natural anatomical (coronal and horizontal) structures of the lower lumbar facet joints limit the flexion and extension movements of the spine to reduce the mechanical forces [16,25]. Second, it has been documented that spinal flexion and extension cause an increase in intradiscal pressure [26,27]. Third, during lumbar extension, particularly if the disk height is decreased, the ends of the inferior articular processes may come into contact with the pars interarticularis and lamina, which is thought to cause pain [29]. These stimuli could be biomechanical initiators of foraminal osteophytic processes [3].

According to Dunlop et al. [30], significant loads are transmitted to the facets more effectively than to the pars interarticularis. According to their findings, the pars interarticularis, one of the load-bearing regions, is the anatomical region where osteophytes originate from the pedicle–lamina junction described in our study (Figure 3A,B). According to Prasad et al. [31], hyperextension of the spine increases the load on the facet joints. Many studies have found that lumbar extension may cause pain [30,32]. Moreover, Yang and King [19] found that further overloading of the facet joints altered the anatomical orientation of the facets. As the disk height decreases, the tip of the inferior articular processes makes contact with the surrounding bone tissue during forward flexion [29]. Several authors have reported that the normal compressive loads carried by the facets increase with unrestricted (above physiological limits) lumbar flexion movement [33,34,35]. These concepts contribute to a better understanding of the process that causes mechanically induced lumbar foraminal osteophytes. The movement segment becoming more immobile supports our claim that it serves as a protective mechanism [29,30,32,33,34,35]. However, further studies with high levels of evidence are required to verify this. Atul Goel suggests that the formation of osteophytes around the facet joint may have a protective rather than a harmful or pathological effect [28].

### 4.3. Osteophytes and Foraminal Stenosis

Lumbar foraminal stenosis is one of the causes of pain associated with radiculopathy [36]. Foraminal stenosis can be caused by various factors, including disk protrusion, decreased disk height, facet hypertrophy, and osteophytes on the vertebral endplates [37]. Lee et al. [37] developed a grading system for foraminal stenosis. When evaluating foraminal stenosis, morphological parameters, such as foraminal height, superior foraminal width, middle foraminal width, minimum foraminal height, pedicle length, and posterior disk height, must be considered [38]. The osteophytes described in this study provide visual evidence of stenosis in the foraminal volume (Figure 1, Figure 2, Figure 3 and Figure 4). Identifying these osteophytes before surgery has the potential to improve surgical outcomes. Foraminal pathologies are among the leading causes of failed back surgery syndrome. Furthermore, the osteophytes described in the present study (Figure 1, Figure 2, Figure 3 and Figure 4) will improve preoperative assessment and awareness. This will help to explain some clinical findings observed after surgery. Our results provide surgeons with a therapeutic target within a novel framework.

In a recent and significant study, Murata et al. [39] linked insufficient decompression of the vertebral osteophyte and intervertebral disc complex (O/D complex) to poor surgical outcomes. Murata et al. specifically recommend removing osteophytes from the lower endplates of the upper vertebrae (Figure 2A–D) for foraminal decompression. Moreover, Murata et al. identified the osteophyte size (O/D complex) as a predictor of recovery. They emphasize the importance of osteophyte size in treating back and leg pain. However, Murata et al. have only focused on one type of osteophyte (originating from the lower endplate of the upper vertebrae). Despite the excision of a single osteophyte, the 2- and 5-year outcomes are quite successful [39]. In our study, we found four osteophytes that narrow the foramen (Figure 1, Figure 2, Figure 3 and Figure 4). The osteophytes we described (Figure 1, Figure 2, Figure 3 and Figure 4) could help surgeons perform foraminal decompression [39]. Murata et al. emphasize that the structure known as an osteophyte, which originates from the lower endplates of the upper vertebrae, is also an important factor in patient selection and surgical planning. Therefore, the four osteophytes we identified (Figure 1, Figure 2, Figure 3 and Figure 4) may be important in patient selection and surgical decision making. Like Murata et al., Atul Goel [24] observes that osteophytes narrow the lumbar intervertebral foramen. Our visual findings show that osteophytes significantly narrow the lumbar intervertebral foramen; these findings are consistent with those of previous studies (Figure 1, Figure 2, Figure 3 and Figure 4).

### 4.4. Limitations of this Study

The present study has several limitations. First, there is no precise definition of foraminal osteophytes in the literature. Second, millimetric osteophytes can cause observers to make different observations, which affects the calculation of their incidence. Third, we reconstructed the images that the other branches requested. We assumed this patient group was asymptomatic, but some patients likely experienced symptoms. Fourth, the images in this study were reconstructed, and they may not have met the requirements of the study. The L4–L5 and L5–S1 levels are very common for osteophyte formation but are not limited to these regions. The paper only reported on the L4–L5 and L5–S1 levels. The L1–L2, L2–L3, and L3–L4 foramen could also be investigated. The images were analyzed without the expertise of a radiologist, which may have had an impact on the results of the study. This is because partial volume effects, motion artifacts, or foraminal variations may be misidentified as osteophyte formations. The images used in the study came from two multi-detector CT machines (one with 128 slices and the other with 16 slices). Despite attempts to control for confounding variables, the inherent observational nature of the study may have allowed residual confounding factors to persist. Future studies across multiple centers must confirm our findings in diverse patient populations.

## 5. Conclusions

This study presented the distribution and morphology of foraminal osteophytes in the sagittal plane in a large number of patients. We identified four types of osteophytes in the L4–L5 and L5–S1 foramina and discussed their potential effects on osteoarthritis, biomechanics, and foraminal stenosis. Awareness of foraminal osteophytes before, during, and after surgery will improve patient management. However, additional research is needed to collect data with high levels of evidence.

## Figures and Tables

**Table 1:** Descriptive values of variables.

Variable	Group	Frequency	Percentage
Gender	Male	697	56.9
	Female	527	43.1
L5–S1	None	1025	83.7
	Left	65	5.3
	Right	57	4.7
	Right and Left	77	6.3
L4–L5	None	1151	94.0
	Left	27	2.2
	Right	27	2.2
	Right and Left	19	1.6
	Total	1224	100.0
Variable	Mean ± SD	Min–Max	
Age	47.75 ± 19.03	18–109	

Mean ± SD, mean ± standard deviation; Min, minimum value; Max, maximum value.

**Table 2:** Comparison between the measurements of osteophyte counts in L5 and L4 foramina.

Variable	Group	n/%	L5–S1		Total	Test Value ^a^	*p*-Value
			Absent	Present			
**L4–L5**	**Absent**	**n**	957	176	1151	**12,094**	<0.001 *
		**%**	95.1%	88.4%	94.0%		
	**Present**	**n**	50	23	73		
		**%**	4.9%	11.6%	6.0%		
**Total**		**n**	**1025**	**199**	**1224**		
		**%**	**100.0%**	**100.0%**	**100.0%**		

n, number of samples; %, percentage; test value ^a^, chi-square test value (*χ*^2^); *p*-value, statistical significance; * *p* < 0.05. There is a statistically significant difference between the groups. Bold numbers (for test value and *p*-value) indicate significant difference.

**Table 3:** Comparison of measurements by gender.

Variable	Group	n/%	Gender		Total	Test Value ^a^	*p*-Value
			Male	Female			
		%	10.1%	10.6%	10.3%		
**L5–S1**	**Absent**	**n**	589	436	1025	3259	0.353
		**%**	84.5%	82.7%	83.7%		
	**Left**	**n**	33	32	65		
		**%**	4.7%	6.1%	5.3%		
	**Right**	**n**	36	21	57		
		**%**	5.2%	4.0%	4.7%		
	**Right and Left** **(Bilateral)**	**n**	39	38	77		
		**%**	5.6%	7.2%	6.3%		
**L4–L5**	**Absent**	**n**	654	497	1151	1837	0.607
		**%**	93.8%	94.3%	94.0%		
	**Left**	**n**	16	11	27		
		**%**	2.3%	2.1%	2.2%		
	**Right**	**n**	18	9	27		
		**%**	2.6%	1.7%	2.2%		
	**Right and Left** **(Bilateral)**	**n**	9	10	19		
		**%**	1.3%	1.9%	1.6%		
**Total**		**n**	**697**	**527**	**1224**		
		**%**	**100.0%**	**100.0%**	**100.0%**		

n, sample size; %, percentage; test value ^a^, chi-square test value (χ^2^); *p*-value. There is a statistically significant difference between the groups. Bold letters and numbers (for test value and *p*-value) indicate significant difference.

**Table 4:** Comparison of measurements by age.

Variable	Group	Mean ± SD	M	Min–Max	Test Value	*p*-Value	Difference
**Left L5–S1**	**Absent**	46.01 ± 18.6	43.00	18–109	**42,068.000** **^a^**	<0.001 *	Present
	**Present**	61.08 ± 16.91	61.00	19–101			
**Right L5–S1**	**Absent**	45.94 ± 18.37	43.00	18–109	**37,973.000** **^a^**	<0.001 *	Present
	**Present**	62.52 ± 17.95	63.00	20–101			
**L5–S1**	**Absent**	45.17 ± 18.22	42.00	18–109	**112,789** **^b^**	<0.001 *	1 and 2, 1 and 3, 1 and 4
	**Left**	58.08 ± 16.36	56.00	19–93			
	**Right**	61.05 ± 19.14	62.00	20–99			
	**Right and Left**	63.61 ± 17.06	64.00	20–101			
**Left L4–L5**	**Absent**	47.00 ± 18.77	44.00	18–109	**11,234.000 ^a^**	<0.001 *	Present
	**Present**	67.07 ± 15.25	66.50	20–92			
**Right L4–L5**	**Absent**	47.09 ± 18.09	44.00	18–109	**12,662.500 ^a^**	<0.001 *	Present
	**Present**	64.7 ± 14.00	62.00	33–92			
**L4–L5**	**Absent**	46.63 ± 18.71	44.00	18–109	**65,559 ^b^**	<0.001 *	1 and 2, 1 and 3, 1 and 4
	**Left**	66.85 ± 16.28	69.00	20–87			
	**Right**	62.81 ± 13.9	63.00	33–87			
	**Right and Left**	67.37 ± 14.07	61.00	38–92			

SD, standard deviation; M, median; Min, the smallest value obtained; Max, the largest value obtained; test value ^a^, Mann–Whitney test; test value ^b^, Kruskal–Wallis test; *p*-value, statistical significance; * *p* < 0.05. There is a statistically significant difference between the groups. Bold letters and numbers (for test value and *p*-value) indicate significant difference.

## Data Availability

The raw data supporting the findings of this study are available from the corresponding authors upon reasonable request.

## Abbreviations

IVF: lumbar intervertebral foramen, SD: standard deviation, Min: minimum, Max: maximum.
